# Valorization of cheese whey to lactobionic acid by a novel strain *Pseudomonas fragi* and identification of enzyme involved in lactose oxidation

**DOI:** 10.1186/s12934-022-01907-0

**Published:** 2022-09-08

**Authors:** Jiawei Wu, Peng Liu, Zhaojuan Zheng, Jia Ouyang

**Affiliations:** 1 Jiangsu Co-Innovation Center of Efficient Processing and Utilization of Forest Resources, College of Chemical Engineering, Nanjing Forestry University, Nanjing, 210037 People’s Republic of China; 2 School of Grain Science and Technology, Jiangsu University of Science and Technology, Zhenjiang, 212003 People’s Republic of China

**Keywords:** Lactobionic acid, Glucose dehydrogenase, Whey, Cell biocatalysis, *Pseudomonas fragi*, Lactose oxidation

## Abstract

**Background:**

Efficient upgrading of inferior agro-industrial resources and production of bio-based chemicals through a simple and environmentally friendly biotechnological approach is interesting Lactobionic acid is a versatile aldonic acid obtained from the oxidation of lactose. Several microorganisms have been used to produce lactobionic acid from lactose and whey. However, the lactobionic acid production titer and productivity should be further improved to compete with other methods.

**Results:**

In this study, a new strain, *Pseudomonas fragi* NL20W, was screened as an outstanding biocatalyst for efficient utilization of waste whey to produce lactobionic acid. After systematic optimization of biocatalytic reactions, the lactobionic acid productivity from lactose increased from 3.01 g/L/h to 6.38 g/L/h in the flask. In batch fermentation using a 3 L bioreactor, the lactobionic acid productivity from whey powder containing 300 g/L lactose reached 3.09 g/L/h with the yield of 100%. Based on whole genome sequencing, a novel glucose dehydrogenase (GDH1) was determined as a lactose-oxidizing enzyme. Heterologous expression the enzyme GDH1 into *P. putida* KT2440 increased the lactobionic acid yield by 486.1%.

**Conclusion:**

This study made significant progress both in improving lactobionic acid titer and productivity, and the lactobionic acid productivity from waste whey is superior to the ever reports. This study also revealed a new kind of aldose-oxidizing enzyme for lactose oxidation using *P. fragi* NL20W for the first time, which laid the foundation for further enhance lactobionic acid production by metabolic engineering.

**Graphical Abstract:**

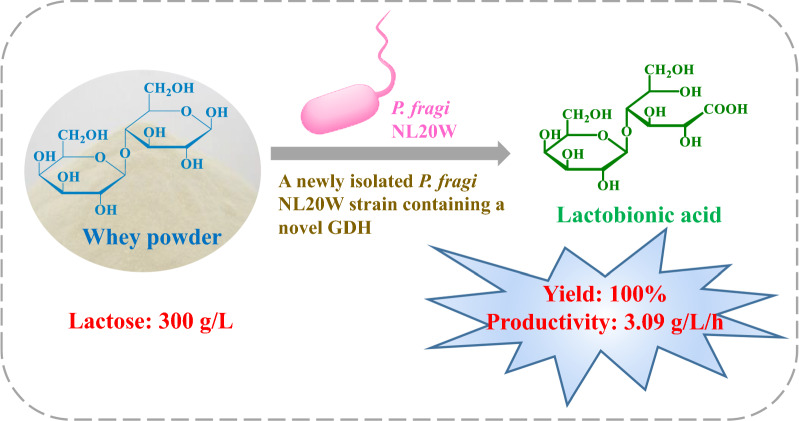

**Supplementary Information:**

The online version contains supplementary material available at 10.1186/s12934-022-01907-0.

## Background

Lactobionic acid (4-*O*-*β*-galactopyranosyl-D-gluconic acid) is a versatile aldonic acid obtained from the oxidation of lactose, with a plethora of applications in the food, tissue engineering, cosmetic, pharmaceutical, and chemical industries, due to its excellent properties, like nontoxic, antioxidant, biocompatible, biodegradable, metal-chelating, and moisturizing properties [1–3]. Lactobionic acid is approved by the U.S. Food and Drug Administration as a food additive, such as an acidulant with a sweet taste, a functional beverage additive and a meat water retention agents [4, 5]. Some studies suggested that the annual intake of lactobionic acid be 760 mg because it occurred in beverages and foods [6]. Commercially available lactobionic acid is primarily manufactured via chemical synthesis. This process is energy-intensive, with toxic, high-cost metals as catalysts, and generates undesirable by-products inevitably [7]. In contrast, biological synthesis has advantage of high selectivity, mild reaction, and non-toxicity, which is a more ideal alternative method. In recent years, production of lactobionic acid using biotechnological routes has been the focus of researchers, who produce lactobionic acid through enzymatic catalysis and microbial bioconversion [8–10]. For the enzymatic process, glucose/fructose dehydrogenase [11], cellobiose dehydrogenase [10], and carbohydrate oxidase [12] had been reported. However, the enzymes are complex to prepare, unstable in industrial environments, and require cofactors to be activated [12]. Compared with enzymes as biocatalysts, microbial cells are more desirable because of its easy preparation, cost-effectiveness, and strong robustness [1].

Many bacteria and fungi show the ability to oxidize lactose into lactobionic acid. Microorganisms belonging to the genera of *Pseudomonas* [4], *Burkholderia* [13, 14], *Acetobacter* [8] and *Zymomonas* [15] have been used for lactobionic acid production. In most cases, the cell bioconversion had lower productivity than the enzymatic catalysis method. Among these bacteria, *P. taetrolens* showed the highest production level (titer of 200 g/L and productivity of 7.41 g/L/h from lactose) [16, 17]. However, to compete with other methods, the lactobionic acid production titer and productivity should be further improved.

An important feature in the production of bio-based chemicals is the utilization of cheap feedstocks as raw materials in bioprocesses. Therefore, during the last few years, there have been several reports to obtain lactobionic acid through the biotechnological pathway with whey as low-cost feedstock [18–20]. In addition to a cheap resource, whey is also a potential pollutant generated in the dairy industry. It is difficult to be disposed of because of its high biochemical oxygen demand and chemical oxygen demand and high annual production. The European Union is the world's largest producer of whey powder, with an annual production of about 1.9 million tons, but only a small portion of whey is processed into powder due to its low commercial value [21]. Apart from water, the main content in whey is lactose (up to 70% by dry weight), which can serve as an ideal substrate to produce lactobionic acid and thus upgrading this inferior raw substrate.

In this study, we aimed to isolate a microorganism to convert lactose into lactobionic acid with high catalytic performance. Various strategies were applied to improve the productivity of lactobionic acid biosynthesis at high substrate concentrations from cheese whey. We also sequenced the entire genome of the isolated strain (*Pseudomonas fragi* NL20W), and based on these informations, a novel pyrroloquinoline quinone-dependent glucose dehydrogenase (PQQ-GDH) was determined as lactose-oxidizing enzyme. These findings expand the current understanding of *P. fragi* NL20W and GDH for aldonic acids biosynthesis and prove the potential industrial applicability of them for lactobionic acid production on a large-scale.

## Results and discussion

### Investigating the catalytic performance of P. fragi NL20W

**Fig. 1:**
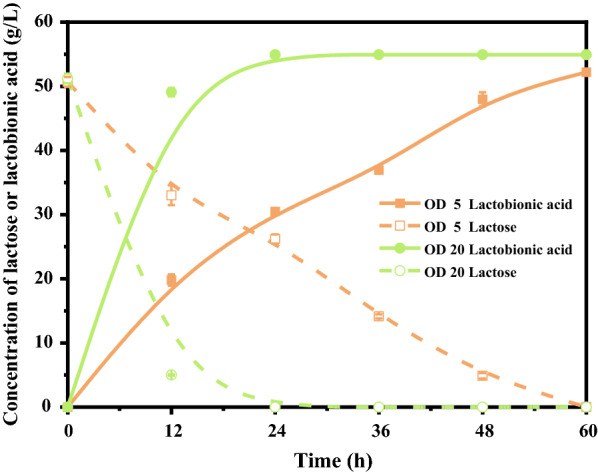
Time courses of lactobionic acid production from lactose by *P. fragi* NL20W in flasks. Reaction conditions: 50 g/L lactose, cell densities of 5 and 20 at OD_600nm_, 7.3 g/L CaCO_3_, 30 °C, pH 7.0, 200 rpm

In order to test the ability of *P. fragi* NL20W to oxidize lactose, reactions were performed using resting cells of *P. fragi* NL20W with different cell concentration (OD_600nm_ 5 and 20, 1.63*10^10^ CFU corresponding to OD_600nm_ 1). As shown in Fig. 1, regardless of the cell dosage, all lactose was oxidized into lactobionic acid as the sole product (Additional file 1: Fig. S1). The catalytic rate at OD_600nm_ 20 was significantly higher than that at OD_600nm_ 5, which consumed all lactose in 24 h and 60 h, respectively.

In previous studies, Mao et al. found that *P. fragi* TCCC11892 had the ability to oxidize lactose into lactobionic acid when investigating the ability of four species of *Pseudomonas* to produce aldonic acids [22]. Beyond that, no *P. fragi* strain was reported as lactobionic acid producer. However, no further study of *P. fragi* TCCC11892 on lactobionic acid production was followed. This research on *P. fragi* NL20W is the first detailed investigation on lactobionic acid production by *P. fragi* species.

### Effect of carbon source on the catalytic performance of P. fragi NL20W

**Fig. 2:**
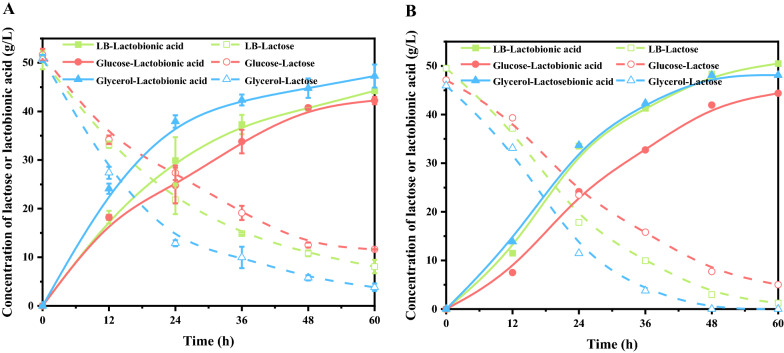
Effect of carbon source on lactobionic acid production. Reaction conditions: **A**, resting cells harvested from different media, 50 g/L lactose, cell densities of 5 at OD_600nm_, 7.3 g/L CaCO_3_, 30 °C, pH 7.0, 200 rpm; **B**, growing cells in different media, initial inoculation of 0.2 at OD_600nm_, other conditions same as A

Although LB medium is rich in carbon and nitrogen resource, which make the strain grow well, we tried to replace it with a cheap medium due to its high price. In a co-fermentation system, Alonso et al. combined cheese whey and glucose or glycerol as co-substrates to investigate their effects on lactobionic acid production patterns [23]. In this study, glucose or glycerol was used as a carbon source supplemented with a trace of yeast extract and mineral salts to determine which medium enabled the highest catalytic activity. *P. fragi* NL20W could grow fast in both glucose and glycerol media, comparable to that in LB medium, but the catalytic performances of cells collected from different media were obviously different. The oxidation rate of cells grown in glycerol medium was significantly faster than that in glucose medium, and even faster than that in LB medium unexpectedly, regardless of using resting cells or growing cells as biocatalysts (Fig. 2). For resting cells, the yield of lactobionic acid obtained from glycerol-cultured cells reached 87.98% at 48 h, which was 11.52% higher than that produced by glucose-cultured cells. For growing cells, the yields of lactobionic acid were 99.75%, 89.51% and 78.43% by glycerol-cultured, LB-cultured, and glucose-cultured cells at 48 h, with lactobionic acid productivity of 0.95 g/L/h, 0.92 g/L/h, and 0.77 g/L/h, respectively. The consumption rate of lactose was faster than that in the resting cells catalytic system. Overall, the results for cell growth and lactobionic acid production indicated that glycerol was a better carbon source, which was used in the following experiments.

### Production of lactobionic acid from lactose by resting cell in the flask

**Fig. 3:**
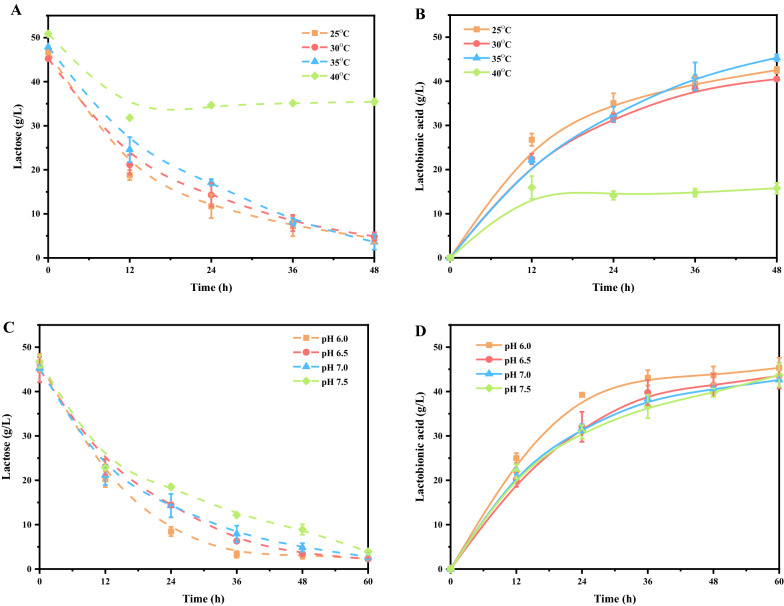
Effects of temperature (**A**, **B**) and pH (**C**, **D**) on lactobionic acid production. Reaction conditions: **A**, 50 g/L lactose, cell densities of 5 at OD_600nm_, 7.3 g/L CaCO_3_, temperature in the range of 25 °C–40 °C, pH 7.0, 200 rpm; **B**, same as **A**; **C**, 50 g/L lactose, cell densities of 5 at OD_600nm_, 7.3 g/L CaCO_3_, 30 °C, pH in the range of 6.0–7.5, 200 rpm; **D**, same as A

The biocatalytic conditions including pH, temperature, and metal ions were commonly surveyed to determine their effects on lactobionic acid production [4, 20, 24, 25]. In order to further increase the lactobionic acid productivity of *P. fragi* NL20W, these parameters were studied in detail. When the temperature was raised from 25 °C to 35 °C, there were no obvious differences in lactobionic acid yield and productivity. However, when the temperature was further increased to 40 °C, lactose oxidation occurred only in the initial 12 h, with final lactobionic acid yield of 29.62%. The concentration of lactose remained constant after 12 h, which indicated high temperature inhibiting the oxidative activity of *P. fragi* NL20W toward lactose (Fig. 3A, B). With the increase of pH from 6.0–7.5, the lactose oxidation ability of *P. fragi* NL20W substantially decreased. In the first 12 h, pH had little effect on the catalytic performance of *P. fragi* NL20W. In contrast, from 12 to 36 h, it could be obviously found that the catalytic efficiency was much higher under pH 6.0 (Fig. 3C, D). At pH 6.0, lactose was almost exhausted after 36 h, with lactobionic acid productivity of 1.20 g/L/h, which suggested the lactose oxidative activity of *P. fragi* NL20W favored slightly acidic conditions.

**Fig. 4:**
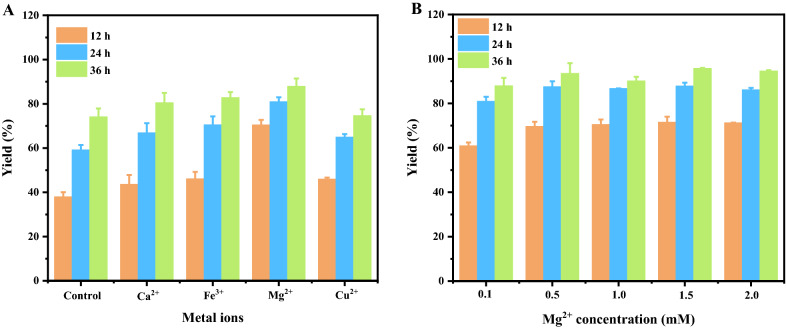
Effects of metal ions on lactobionic acid production. Reaction conditions: **A**, 50 g/L lactose, cell densities of 5 at OD_600nm_, 7.3 g/L CaCO_3_, with or without designated metal ions at 1 mM, 30 °C, pH 7.0, 200 rpm; **B**, Mg^2+^ in the range of 0.1 mM–2.0 mM, other conditions same as **A**

**Fig. 5:**
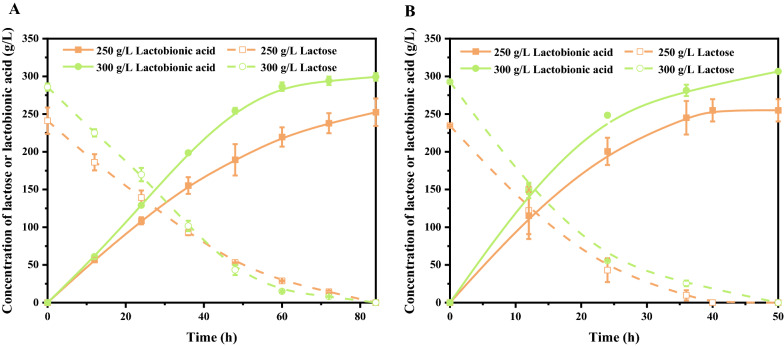
Comparison of lactobionic acid production before and after optimization. Reaction conditions: **A** lactose of the designated concentration, cell density of 20 at OD_600nm_, CaCO_3_ with half molar ratio of lactose, 30 °C, pH 7.0, 200 rpm; **B** pH 6.0, 0.5 mM Mg^2+^, other conditions same as **A**

Figure 4A showed the effect of metal ions on lactobionic acid production. It was found that all examined metal ions, including Ca^2+^, Fe^3+^, Mg^2+^, and Cu^2+^ had positive effects on lactose oxidation. Among them, Mg^2+^ played the most significant role, with lactobionic acid yield increased by 85% at 12 h compared to the control. Although other metal ions could also promote the production of lactobionic acid, but their effects were not as positive as Mg^2+^. Next, the optimal concentration of Mg^2+^ ranging from 0.1 mM to 2.0 mM was further investigated. When the concentration of Mg^2+^ varied from 0.1 mM to 0.5 mM, the yields of lactobionic acid at 36 h increased from 87.93% to 93.54%, but the yields did not further increase at higher Mg^2+^ concentration (Fig. 4B). Finally, the catalytic performance of *P. fragi* NL20W before and after optimization was evaluated and compared under high lactose concentration. As shown in Fig. 5, lactose was basically converted until 84 h before optimization, while 250 g/L lactose could be completely converted at 40 h and 300 g/L lactose at 84 h after optimization. The productivities were increased from 3.01 g/L/h to 6.38 g/L/h at 250 g/L, and 3.56 g/L/h to 6.25 g/L/h at 300 g/L, indicating the effectiveness of the combined optimal reaction conditions.

### Production of lactobionic acid from whey powder by growing cell in the bioreactor

**Fig. 6:**
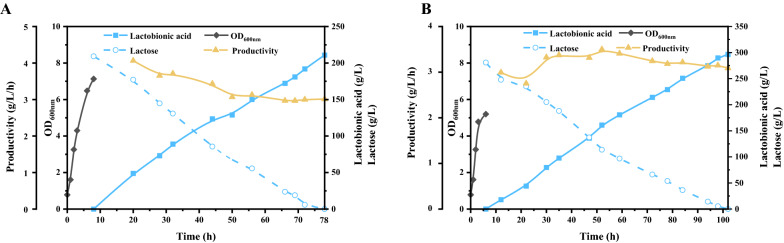
Lactobionic acid production from whey powder in 3 L bioreactor. Reaction conditions: **A**, whey powder containing 200 g/L lactose added at 8 h, initial inoculation of 1 at OD_600nm_, 30 °C; **B**, whey powder containing 300 g/L lactose added at 6 h, other conditions same as A

To further investigate the performance of *P. fragi* NL20W and elevate the cost effectiveness, batch cultivation experiments were carried out in a 3 L bioreactor with whey powder as substrate under controlled conditions. Glycerol was still adopted as carbon source to facilitate cell growth and lactose conversion. Two different strategies were undertaken to evaluate the effect of whey powder adding time on lactobionic acid production. When whey powder was added simultaneously with cell inoculation, a total of 200 g/L lactose could be completely converted in 87 h with an average productivity of 1.62 g/L/h (Additional file 1: Fig. S2). Due to the turbidity of the whey components, the optical density of the strain could not be accurately measured. However, the unspecified components in whey or other harmful effects including high osmolality might influence the cell growth of *P. fragi* NL20W, and render the reaction rate sluggish. Therefore, we also determined the lactobionic acid production profiles when whey powder was added at the middle and late logarithmic phase. In any case, the productivities of lactobionic acid could be enhanced to a large extent. In the case that the whey powder containing 200 g/L lactose was added at 8 h (late logarithmic phase), and the yield of lactobionic acid reached 100% at 78 h with average productivity of 3.01 g/L/h (Fig. 6A), which was 85.80% higher than that of adding whey powder at 0 h. We also tried to increase the lactose concentration in whey to 300 g/L. In this case, the whey powder was added at 6 h (middle logarithmic phase), and all lactose could be quickly converted into lactobionic acid in 102 h with a yield of 100% and average productivity of 3.09 g/L/h (Fig. 6B). Although the lactobionic acid productivities using whey powder as substrate were lower than that using pure lactose, the results in this study outperformed the best reports with *P. taetrolens* as biocatalyst (Table 1). Compared with previous studies, this study made significant progress both in improving lactobionic acid titer and productivity. These results also demonstrated the new isolated *P. fragi* NL20W strain had great application potential in the valorization of waste cheese whey to lactobionic acid.

**Table 1:** Comparison of Research on Microbial Production of Lactobionic Acid

Microorganism	Production mode	Biocatalyst	Substrate	Substrate titer (g/L)	Productivity (g/L/h)	Yield (%)	Reference
*A. orientalis*	batch in flask	resting cell	lactose	49	0.54	98	[8]
*B. cepacia* no.24	fed-batch in flask	growing cell	lactose	400	1.67	100	[13]
*B. cepacia* no.24^a^	fed-batch in flask	resting cell	lactose	150	5.55	100	[14]
*K. medellinensis*	batch in flask	resting cell	lactose	–	–	–	[26]
*Pseudomonas* sp. LS131	fed-batch in bioreactor	growing cell	lactose	290	1.87	90	[27]
*Zymomonas mobilis*^b^	batch in flask	permeabilized cells	lactose, fructose	182	7.60	78	[15]
*P. taetrolens*	batch in bioreactor	growing cell	concentrate whey	78	1.63	100	[18]
*P. taetrolens*	fed-batch in bioreactor	growing cell	concentrate whey	164	2.05	82	[24]
*P. taetrolens*^c^	batch in bioreactor	growing cell	concentrate whey	200	2.11	100	[17]
*P. taetrolens*^c^	batch in bioreactor	growing cell	lactose	200	8.70	100	[17]
*E. coli*^d^	batch in flask	growing cell	concentrate whey	200	0.62	100	[28]
*P. fragi* NL20W	batch in flask	resting cell	lactose	300	6.25	100	This study
*P. fragi* NL20W	batch in bioreactor	growing cell	whey powder	200	3.01	100	This study
*P. fragi* NL20W	batch in bioreactor	growing cell	whey powder	300	3.09	100	This study

^a^Mutant strain

^b^Permeabilized cells

^c^Recombinant *P. taetrolens* strain with homologous expression of quinoprotein glucose dehydrogenase gene.

^d^Recombinant *E. coli* strain with homologous expression of malate quinone oxidoreductase

### Identification of enzyme involved in lactose oxidation

After demonstrating that *P. fragi* NL20W was a good biocatalyst for conversion of lactose and cheese whey to lactobionic acid, we expected to better understand the enzyme involved in this oxidation reaction. Presently, many kinds of lactose-oxidizing enzymes had been identified from both fungal and bacteria. For bacteria, such lactose-oxidizing enzymes included PQQ-dependent glucose dehydrogenase [26], glucose-fructose oxidoreductase [29], and malate:quinone oxidoreductase [28]. In this research, based on genome annotation and sequence alignment, six candidate enzymes of *P. fragi* NL20W were selected, including four PQQ-dependent glucose dehydrogenases and two malate:quinone oxidoreductases (Additional file 1: Table S1). Their functions in lactose oxidation were assessed through heterologous overexpression in *P. putida* KT2440, which innately had poor ability to convert lactose into lactobionic acid.

**Fig. 7:**
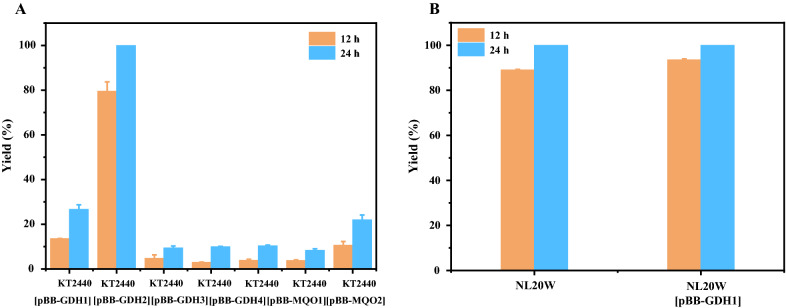
Yields of lactobionic acid from lactose by different strains. **A**, wild-type *P. putida* KT2440 and six derivatives; **B**, wild-type *P. fragi* NL20W and one derivative. Reaction conditions: 50 g/L lactose, cell densities of 20 at OD_600nm_, 7.3 g/L CaCO_3_, 30 °C, pH 7.0, 200 rpm

The four GDH genes and two MQO genes were cloned into the expression vector pBBR1MCS2, resulting in 6 different plasmids, which were individually transformed into *P. putida* KT2440 to examine their roles in the synthesis of lactobionic acid. As shown in Fig. 7A, although wild-type *P. putida* KT2440 could convert lactose into lactobionic acid to some extent, the conversion rate was rather low, with yields of 13.57% and 26.69% after 12 h and 24 h, respectively. By contrast, the derivative strain overexpressing GDH1 showed significantly increased yields, at 79.54% and 100% after 12 h and 24 h, respectively. The lactobionic acid yield increased by 486.1%. These results were almost as good as those obtained by *P. fragi* NL20W. However, the other 5 candidate genes gave detrimental effects on lactose conversion, presumably due to the additional burden caused by exogenous plasmids. Furthermore, we performed homologous expression of GDH1 gene in *P. fragi* NL20W, and the lactobionic acid yields of 12 h slightly increased from 89.07% to 93.54% (Fig. 7B). As wild-type *P. fragi* NL20W was outstanding enough, further strengthen of lactobionic acid production might require more than just enzyme overexpression. Other strategies should also be considered, such as balanced coexpression of GDH and cofactor PQQ, sufficient oxygen supply, etc. Anyway, the GDH1 from *P. fragi* NL20W was identified as a novel lactose-oxidizing enzyme of *P. fragi.*

We performed amino acid sequence analysis of GDH1 from *P. fragi* NL20W, which was predicted to be a membrane-bound PQQ glucose dehydrogenase (http://harrier.nagahama-i-bio.ac.jp/sosui/sosui_submit.html). Glucose dehydrogenases harboring PQQ are widely distributed in *Pseudomonas* sp., which participate in glucose metabolism to oxidize glucose into gluconic acid. Among them, the PQQ-dependent glucose dehydrogenase from *P. putida* KT2440 is the most scientifically and industrially attractive [30]. Although it exhibits high catalytic efficiency upon glucose, its narrow substrate specificity limits the application in lactose oxidation. In addition to this, the PQQ-dependent glucose dehydrogenase from *P. taetrolens* had been reported to convert lactose into lactobionic acid [17]. In terms of substrate specificity, the GDH from *P. taetrolens* varied significantly with that of our research. For example, the GDH from *P. taetrolens* had no activity towards arabinose [17], but the GDH from *P. fragi* NL20W did (data not shown), which also indicated the GDH in this study is a new kind of aldose-oxidizing enzyme.

## Conclusion

In conclusion, a new strain identified as *P. fragi* with high lactobionic acid production ability was isolated and its potential in the upgrading of cheese whey was fully exploited. An efficient approach was developed for improving lactobionic acid titer and yield even the substrate up to 300 g/L. In addition, scale-up synthesis was realized in the bioreactor, which laid the foundation for large-scale industrial production processes. It is of interest to uncover a novel membrane-bound GDH (GDH1) playing a pivotal role in lactose oxidation. Further study on the substrate spectrums of *P. fragi* NL20W and GDH1 might reveal their advantages in the production of other aldonic acids.

## Materials and methods

### Isolation and genome sequencing of P. fragi NL20W

*P. fragi* NL20W was isolated from soil samples obtained from Purple Mountain (Nanjing, China). Based on the analysis of 16S ribosomal RNA and phylogenetic tree (Additional file 1: Fig. S3), strain NL20W was identified as *P. fragi.* Whole-genome sequencing of *P. fragi* NL20W was also performed and submitted to GenBank under accession No. CP064354.1.

### Microorganism, medium, and growth condition

*P. fragi*, *P. putida* and *Escherichia coli* strains (Additional file 1: Table S2) were grown and proliferated in Luria–Bertani (LB) broth at 30 °C and 37 °C, respectively, with 200 rpm shaking for 12 h. If required, 50 µg mL^−1^ kanamycin was added to the medium to avoid loss of the plasmid. All used solid media contained 15 g L^−1^ agar.

To replace LB medium with a cheap medium, glucose mineral medium and glycerol mineral medium were used. The medium consisted of 5.0 g/L glucose or glycerol, 5.0 g/L yeast extract, 3.4 g/L Na_2_HPO_4_, 1.5 g/L KH_2_PO_4_, 0.25 g/L NaCl, 0.5 g/L NH_4_Cl, 0.52 g/L MgSO_4_, and 2.5 mL/L A9 solution, with pH 7.0. A9 solution was composed of the following: H_3_BO_3_ 300 mg/L, ZnCl_2_ 50 mg/L, MnCl_2_·4H_2_O 30 mg/L, CoCl_2_ 200 mg/L, CuCl_2_·2H_2_O 10 mg/L, NiCl_2_·6H_2_O, 20 mg/L, and Na_2_MoO_4_·2H_2_O 30 mg/L.

### Lactobionic acid production by resting cells in the flask

To examine the effect of carbon resource on lactose catalytic performance of *P. fragi* NL20W, overnight culture in LB medium was spun by centrifugation (12000 g, 25 °C, 5 min), and transferred to fresh LB medium, glucose mineral medium and glycerol mineral medium, respectively, with a starting OD_600nm_ of 0.2. After incubating at 30 °C and 200 rpm for 12 h, cells were harvested by centrifugation (8000 g for 10 min) and washed with 200 mM phosphate buffer (pH 7.0) three times. Biotransformation experiments were conducted in 250 mL flasks containing 10 mL of resting cells with optical density of 5 at OD_600nm_, 50 g/L lactose, and 7.3 g/L CaCO_3_ in 200 mM phosphate buffer (pH 7.0) at 30 °C and 200 rpm.

To investigate the effect of temperature on lactobionic acid production, biotransformation experiments were carried out at 25 °C, 30 °C, 35 °C, and 40 °C, respectively, while other reaction conditions remained the same. To investigate the effect of pH on lactobionic acid production, biotransformation experiments were carried out at pH 6.0, 6.5, 7.0, and 7.5, respectively, while other reaction conditions remained the same. Effect of metal ions, including Ca^2+^, Mg^2+^, Fe^3+^, and Cu^2+^, on lactobionic acid production was tested at the final concentrations of 1 mM, and the effect of Mg^2+^ concentration was evaluated from 0.1 mM to 2.0 mM. Other reaction conditions remained the same.

### Lactobionic acid production by growing cells in the flask

Overnight culture in LB medium was spun by centrifugation (12000 g, 25 °C, 5 min), and transferred to fresh LB medium, glucose mineral medium and glycerol mineral medium, respectively, with a starting OD_600nm_ of 0.2. All media also contained 50 g/L lactose and 7.3 g/L CaCO_3_. Cell growth and lactose bioconversion were accomplished 30 °C and 200 rpm.

### Fermenter condition

Batch fermentation was carried out in a 3 L bioreactor (BXBio, Shanghai, China) at a working volume of 1 L. *P. fragi* NL20W was pre-cultured in glycerol mineral medium, spun by centrifugation and transferred to fresh glycerol mineral medium with a starting OD_600nm_ of 1. Whey powder was directly added to the medium, making lactose with final concentration of 200 g/L or 300 g/L. Bioreactor experiments were conducted with an agitation rate of 350 rpm, and an aeration rate of 1.0 vvm. Excessive foam formation was prevented by the addition of a drop of defoamer. During lactobionic acid production, pH was maintained at 6.0 via automatic addition of 25% NaOH.

### Plasmid and strain constructions

To express GDH1 (QPC34881.1), GDH2 (QPC33644.1), GDH3 (QPC33554.1), GDH4 (QPC37981.1), MQO1 (QPC37714.1), and MQO2 (QPC35030.1) encoding genes from *P. fragi* NL20W, pBBR1MCS2 was used as the expression vector. Oligonucleotide primers used were listed in Table S3. pBBR1MCS-GDH1, pBBR1MCS-GDH2, pBBR1MCS-GDH3, pBBR1MCS-GDH4, pBBR1MCS-MQO1, and pBBR1MCS-MQO2 were constructed by replacing the lacZα fragment of pBBR1MCS2 with GDH and MQO genes, respectively, using the pEASY^®^-Basic Seamless Cloning and Assembly Kit (TransGen Biotech, Beijing, China). The resultant plasmids were individually transformed into *P. fragi* NL20W and *P. putida* KT2440 via electroporation at 2400 V, 200 Ω, and 25 μF.

### Analytical methods

Bacterial growth was measured using a spectrophotometer at a wavelength of 600 nm (OD_600nm_). Lactobionic acid and lactose contents of culture samples were measured by high-performance liquid chromatography (Agilent 1100 series, USA) equipped with a Coregel ION 300 column (Concise Separations, USA) and a refractive index detector (Shimadzu, Japan). The column was eluted with 0.5 mM H_2_SO_4_ at a flow rate of 0.4 mL/min with the column temperature set at 75 °C.

The lactobionic acid yield was calculated as follows:

$$\text{Lactobionic} \; \text{acid} \; \text{yield} = \frac{\text{moles}\; \text{of}\; \text{produced}\; \text{lactobionic}\; \text{acid}}{\text{moles}\; \text{of}\; \text{added}\; \text{lactose}}\times \text{100\%}$$

## Supplementary Information

**Additional file 1: Figure S1. A** Chromatogram of commercial lactose. **B** Chromatogram of commercial lactobionic acid. **C** Chromatogram of lactose and lactobionic acid in the sample. **Figure S2** Lactobionic acid production from whey powder in 3 L bioreactor. Reaction conditions: whey powder containing 200 g/L lactose added at the beginning, initial inoculation of 1 at OD_600nm_, 30 °C. **Figure S3. **Phylogenetic tree derived from 16S rDNA sequence of different strains based on the Neighbor-Joining method. Bootstrap values are given on each branch. Strain NL20W clearly clusters with other strains in the so-called *P. fragi* lineage. **Table S1 **BLAST results of GDHs and MQOs from *P. fragi* NL20W with reported lactose-oxidizing enzymes. **Table S2 **Strains and plasmids used in this study. **Table S3 **Oligonucleotide primers used in this study.

## Data Availability

The datasets supporting the conclusions of this article are included within the article.

## Abbreviations

GDH: Glucose dehydrogenase
PQQ: Pyrroloquinoline quinone
LB: Luria–bertani
OD: Optical density
