# Supplementary material for: Valorization of cheese whey to lactobionic acid by a novel strain Pseudomonas fragi and identification of enzyme involved in lactose oxidation
Source: Microb Cell Fact. 2022 Sep 8;21:184. doi: 10.1186/s12934-022-01907-0 (PMC9461264; doi:10.1186/s12934-022-01907-0)
Supplement: Supplementary file 1 — Additional file 1: Figure S1. A Chromatogram of commercial lactose. B Chromatogram of commercial lactobionic acid. C Chromatogram of lactose and lactobionic acid in the sample. Figure S2 Lactobionic acid production from whey powder in 3 L bioreactor. Reaction conditions: whey powder containing 200 g/L lactose added at the beginning, initial inoculation of 1 at OD600nm, 30 °C. Figure S3. Phylogenetic tree derived from 16S rDNA sequence of different strains based on the Neighbor-Joining method. Bootstrap values are given on each branch. Strain NL20W clearly clusters with other strains in the so-called P. fragi lineage. Table S1 BLAST results of GDHs and MQOs from P. fragi NL20W with reported lactose-oxidizing enzymes. Table S2 Strains and plasmids used in this study. Table S3 Oligonucleotide primers used in this study. [file 12934_2022_1907_MOESM1_ESM.docx]

**Isolation and screening of *Pseudomonas fragi* NL20W**

Soils from 15 different locations at the Purple Mountain (Nanjing, China) were collected for strain screening. Approximately 1 g of each was mixed with 9 mL of sterile saline and an aliquot of 500 μL of suspensions was inoculated into 50 mL of LB medium, cultured at 30℃ and 200 rpm for 12 h. After dilution, the enriched cultures were spread on screening medium (lactose, 50 g/L; yeast extract, 5 g/L; tryptone, 10 g/L; NaCl, 10 g/L; agar, 15 g/L; CaCO_3_, 25 g/L), and incubated at 30℃ until colonies appeared. Representative colonies were selected based on colony size and acid production zone. Then the selected colonies were incubated into liquid screening medium (lactose, 50 g/L; yeast extract, 5 g/L; tryptone, 10 g/L; NaCl, 10 g/L; CaCO_3_, 25 g/L) for further screening. Aliquots were withdrawn from the medium at specified time intervals for quantificational analysis of lactose and lactobionic acid. The strain that produced lactobionic acid with the highest titer and productivity, designated as NL20W, was selected for further analysis.

**
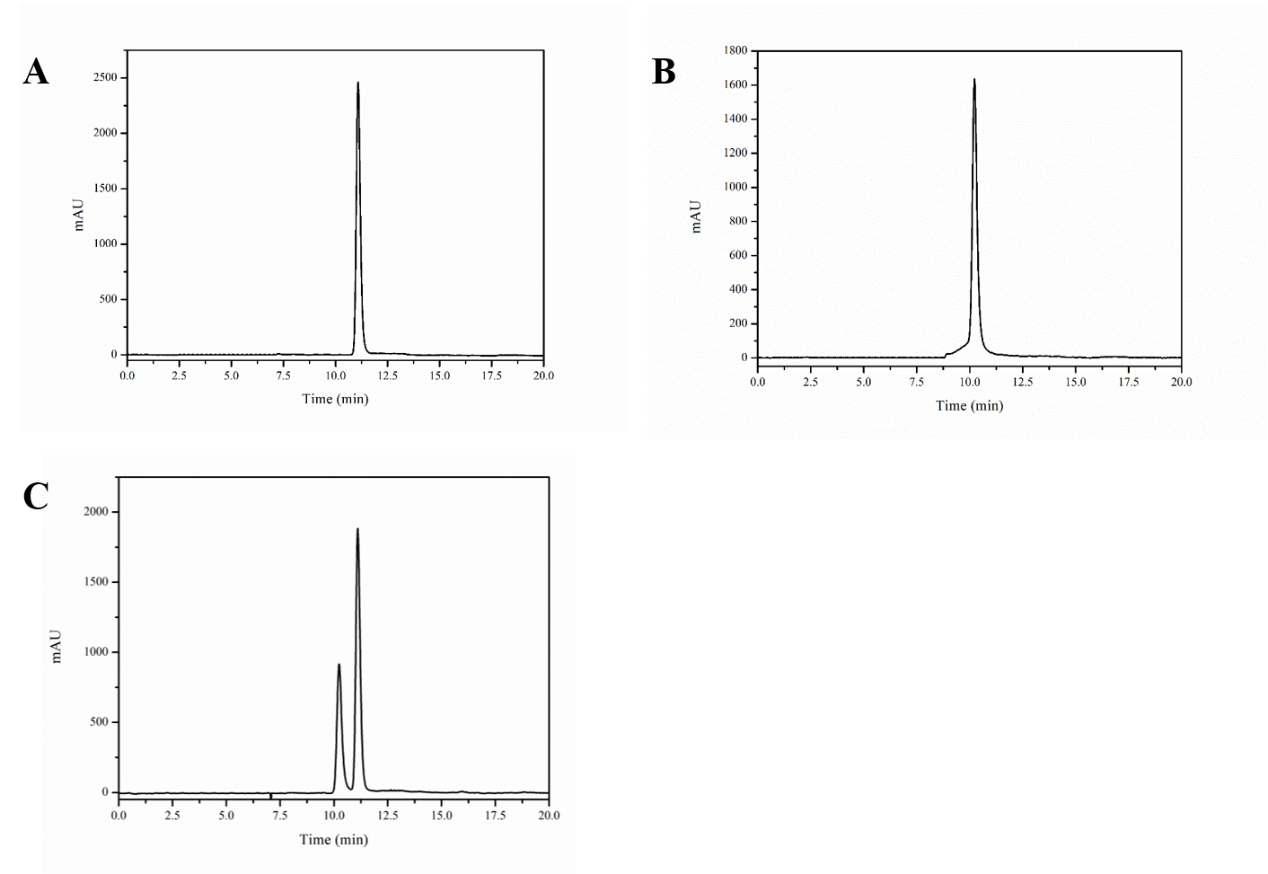
**

**Fig. S1** (A) Chromatogram of commercial lactose. (B) Chromatogram of commercial lactobionic acid. (C) Chromatogram of lactose and lactobionic acid in the sample.

**Fig. S2** Lactobionic acid production from whey powder in 3 L bioreactor. Reaction conditions: whey powder containing 200 g/L lactose added at the beginning, initial inoculation of 1 at OD_600nm_, 30℃.


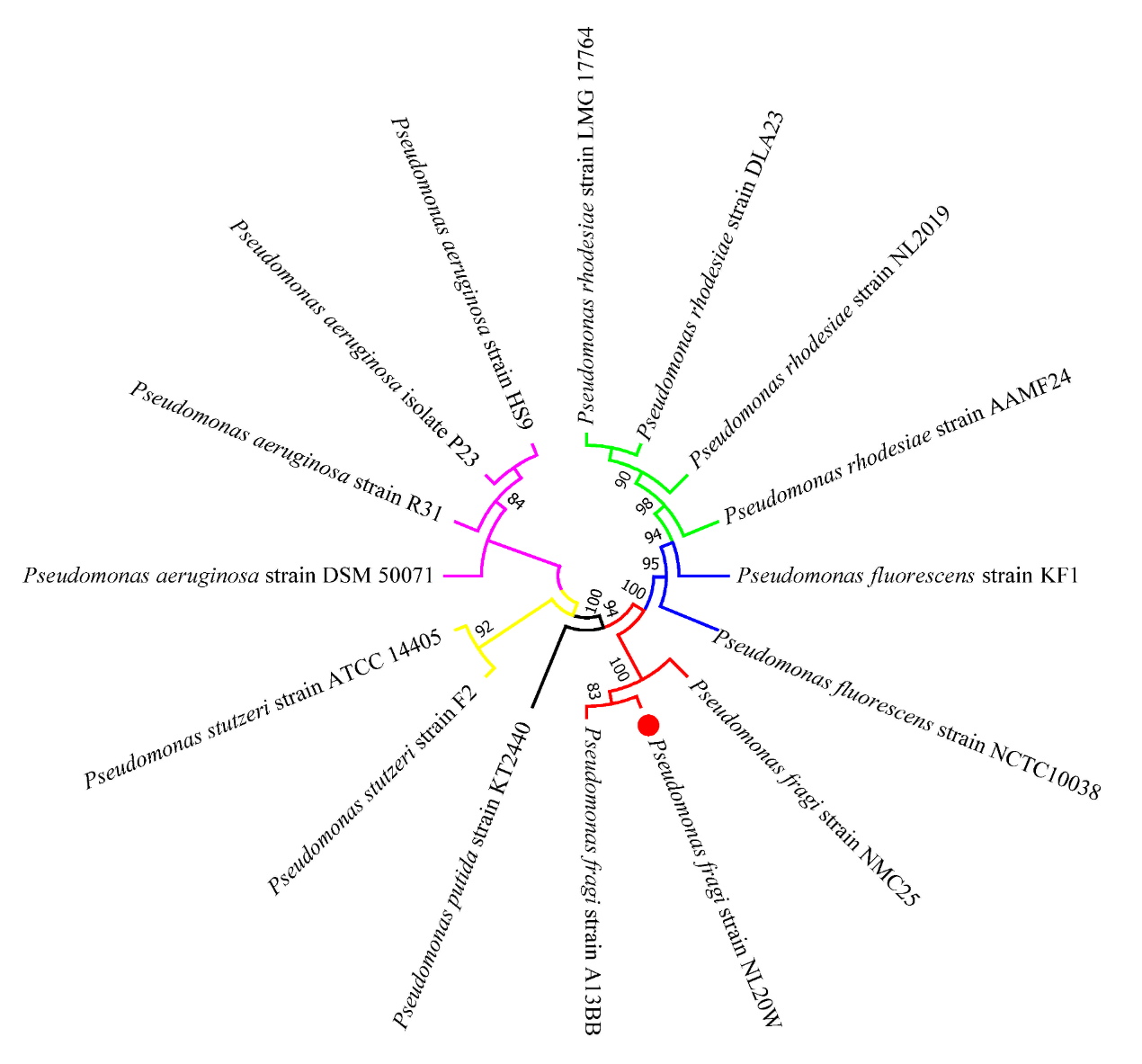


**Fig. S3** Phylogenetic tree derived from 16S rDNA sequence of different strains based on the Neighbor-Joining method. Bootstrap values are given on each branch. Strain NL20W clearly clusters with other strains in the so-called *P. fragi* lineage.

**Table S1 BLAST results of GDHs and MQOs from *P. fragi* NL20W with reported lactose-oxidizing enzymes.**

| *P. fragi* NL20W | Identity / Coverage (%) | | | |
| --- | --- | --- | --- | --- |
|  | GDH from *P. taetrolens* (WP_04838095.1) | GDH from *Acinetobacter calcoaceticus* (5MIN) | GDH from *Komagataeibacter medellinensis* (BAK83009.1) | MQO from *P. taetrolens* (WP_048378186.1) |
| GDH1 (QPC34881.1) | 48.54 / 95 | none | 51.72 / 95 | – |
| GDH2 (QPC33644.1) | 40.68 / 97 | none | 40/100 | – |
| GDH3 (QPC33554.1) | 40.59 / 96 | none | 51.53/95 | – |
| GDH4 (QPC37981.1) | 40.89 / 99 | none | 42.28/96 | – |
| MQO1 (QPC37714.1) | – | – | – | 50.10 / 98 |
| MQO2 (QPC35030.1) | – | – | – | 96.02 / 100 |

**Table S2 Strains and plasmids used in this study.**

| strain and plasmid | characteristic | source or reference |
| --- | --- | --- |
| **strain** | | |
| *P. fragi* NL20W | wild type | this study |
| *P. putida* KT2440 | wild type | this laboratory |
| *E. coli* Trelief^TM^ 5α | cloning host | TsingKe Biotech |
| *P. putida* KT2440[pBB-GDH1] | *P. putida* KT2440 with pBBR1MCS-GDH1 | this study |
| *P. putida* KT2440[pBB-GDH2] | *P. putida* KT2440 with pBBR1MCS-GDH2 | this study |
| *P. putida* KT2440[pBB-GDH3] | *P. putida* KT2440 with pBBR1MCS-GDH3 | this study |
| *P. putida* KT2440[pBB-GDH4] | *P. putida* KT2440 with pBBR1MCS-GDH4 | this study |
| *P. putida* KT2440[pBB-MQO1] | *P. putida* KT2440 with pBBR1MCS-MQO1 | this study |
| *P. putida* KT2440[pBB-MQO2] | *P. putida* KT2440 with pBBR1MCS-MQO2 | this study |
| *P. fragi* NL20W[pBB-GDH1] | *P. fragi* NL20W with pBBR1MCS-GDH1 | this study |
| **plasmid** | | |
| pBBR1MCS2 | constitutive promoter, Kana | this laboratory |
| pBBR1MCS-GDH1 | pBBR1MCS with the full-length GDH1 from *P. fragi* | this study |
| pBBR1MCS-GDH2 | pBBR1MCS with the full-length GDH2 from *P. fragi* | this study |
| pBBR1MCS-GDH3 | pBBR1MCS with the full-length GDH3 from *P. fragi* | this study |
| pBBR1MCS-GDH4 | pBBR1MCS with the full-length GDH4 from *P. fragi* | this study |
| pBBR1MCS-MQO1 | pBBR1MCS with the full-length MQO1 from *P. fragi* | this study |
| pBBR1MCS-MQO2 | pBBR1MCS with the full-length MQO2 from *P. fragi* | this study |

**Table S3 Oligonucleotide primers used in this study.**

| primers | sequence |
| --- | --- |
| GDH1.f | ttactcagccagtttgaacg |
| GDH1.r | atgagcactgaaggtgcttt |
| pBB-GDH1.f | aaagcaccttcagtgctcatagctgtttcctgtgtgaaat |
| pBB-GDH1.r | cgttcaaactggctgagtaagcgttaatattttgttaaaa |
| GDH2.f | ttagttgttgtcaggcaacg |
| GDH2.r | atgaataagcctcaaccttc |
| pBB-GDH2.f | gaaggttgaggcttattcatagctgtttcctgtgtgaaat |
| pBB-GDH2.r | cgttgcctgacaacaactaagcgttaatattttgttaaaa |
| GDH3.f | ttactctttgccaacagcat |
| GDH3.r | atgacggacagtaaacagcc |
| pBB-GDH3.f | ggctgtttactgtccgtcatagctgtttcctgtgtgaaat |
| pBB-GDH3.r | atgctgttggcaaagagtaagcgttaatattttgttaaaa |
| GDH4.f | ttatttttcagcgctattcg |
| GDH4.r | atgacggatgaccatcaatc |
| pBB-GDH4.f | gattgatggtcatccgtcatagctgtttcctgtgtgaaat |
| pBB-GDH4.r | cgaatagcgctgaaaaataagcgttaatattttgttaaaa |
| MQO1.f | ttacagcgccatgtcagccgctggattgct |
| MQO1.r | atgttaaaaaaaatgaacaaggggctactg |
| pBB-MQO1.f | ttgttcattttttttaacatagctgtttcctgtgtgaaat |
| pBB-MQO1.r | cggctgacatggcgctgtaagcgttaatattttgttaaaa |
| MQO2.f | ttacgcgatgctggtattgg |
| MQO2.r | atggcgcataacgaagcagt |
| pBB-MQO2.f | actgcttcgttatgcgccatagctgtttcctgtgtgaaat |
| pBB-MQO2.r | ccaataccagcatcgcgtaagcgttaatattttgttaaaa |
